# Advancing stroke patient care: a network meta-analysis of dysphagia screening efficacy and personalization

**DOI:** 10.3389/fneur.2024.1380287

**Published:** 2024-08-06

**Authors:** Youli Jiang, Yue Chi, Rongjia Pan, Dongqi Zhang, Suzhen Huang, Hao Ju, Yanfeng Li

**Affiliations:** ^1^Department of Neurology, People’s Hospital of Longhua, Shenzhen, China; ^2^Department of Rehabilitation Medicine, Nanjing Drum Tower Hospital, Affiliated Hospital of Medical School, Nanjing University, Nanjing, Jiangsu, China; ^3^Department of Endocrinology, People’s Hospital of Longhua, Shenzhen, China

**Keywords:** dysphagia, swallowing disorders, deglutition, GUSS, MASA

## Abstract

**Introduction:**

The increasing incidence of stroke globally has led to dysphagia becoming one of the most common complications in stroke patients, with significant impacts on patient outcomes. Accurate early screening for dysphagia is crucial to avoid complications and improve patient quality of life.

**Methods:**

Included studies involved stroke-diagnosed patients assessed for dysphagia using bedside screening tools. Data was sourced from Embase, PubMed, Web of Science, Scopus, and CINAHL, including publications up to 10 December 2023. The study employed both fixed-effect and random-effects models to analyze sensitivity, specificity, positive predictive value (PPV), and Negative Predictive Value (NPV), each with 95% confidence intervals. The random-effects model was particularly utilized due to observed heterogeneity in study data.

**Results:**

From 6,979 records, 21 studies met the inclusion criteria, involving 3,314 participants from 10 countries. The analysis included six assessment tools: GUSS, MASA, V-VST, BSST, WST, and DNTA, compared against gold-standard methods VFSS and FEES. GUSS, MASA, and V-VST showed the highest reliability, with sensitivity and specificity rates of 92% and 85% for GUSS, 89% and 83% for MASA, respectively. Heterogeneity among studies was minimal, and publication bias was low, enhancing the credibility of the findings.

**Conclusion:**

Our network meta-analysis underscores the effectiveness of GUSS, MASA, and V-VST in dysphagia screening for stroke patients, with high sensitivity and specificity making them suitable for diverse clinical settings. BSST and WST, with lower diagnostic accuracy, require more selective use. Future research should integrate patient-specific outcomes and standardize methodologies to enhance dysphagia screening tools, ultimately improving patient care and reducing complications.

**Systematic review registration:**

https://www.crd.york.ac.uk/PROSPERO/#recordDetails.

## Introduction

In recent years, the incidence of stroke has increased year by year and has become a major disease that seriously endangers national health worldwide (1). Particularly in stroke patients, dysphagia has distinct characteristics compared to dysphagia resulting from other causes. A study highlighted that 56.6% of acute stroke patients presented with dysphagia, which was significantly associated with older age, greater stroke severity, and larger lesion volumes (2). Dysphagia after stroke can lead to severe complications such as aspiration pneumonia, dehydration, and malnutrition, which in turn may result in prolonged hospitalization, increased likelihood of readmission after discharge, and heightened risk of death (3). Early screening for dysphagia after stroke using accurate and appropriate assessment tools can help to identify potential risk factors early and avoid related complications, thereby improving patient outcomes (4). Accurate diagnosis and management of dysphagia are crucial for preventing these complications and improving the quality of life for stroke patients.

In the current landscape of post-stroke dysphagia evaluation, the primary methods encompass instrumental examinations and clinical scale assessments. The Video fluoroscopic Swallowing Study (VFSS) is widely recognized as the “gold standard” in dysphagia diagnosis, providing dynamic imaging under X-ray to meticulously analyze the swallowing movements of the mouth, pharynx, larynx, and esophagus (5, 6). Another notable method, the Fiberoptic Endoscopic Evaluation of Swallowing (FEES), enables direct observation of the nasal, pharyngeal, and laryngeal structures during natural breathing, coughing, speaking, and swallowing activities (7). Yet, it is crucial to acknowledge the inherent limitations of VFSS, including radiation exposure risks and the potential for aspiration during the procedure (8). Additionally, its dependency on specific person environmental factors restricts its widespread application, with the method’s high costs further impeding its utility for extensive clinical screening. Both VFSS and FEES are acclaimed for their high accuracy (9). They necessitate specialized equipment and trained professionals, which limits their accessibility in certain patient groups. Specifically, VFSS is not recommended for pregnant women and children due to radiation concerns and is advised against frequent use in general patients (10).

Non-instrumental bedside assessment tools remain the primary method for screening and diagnosing dysphagia in clinical settings, particularly for early evaluation of swallowing disorders. Currently, common swallowing disorder assessment tools include water swallowing test, TOR-BSST©, MASA and GUSS, etc. (11–15). Various studies have shown that the accuracy of swallowing function assessment scales varies, with the Gugging Swallow Screen (GUSS) having a sensitivity of 97% and specificity of 67%, the Mann Assessment of Swallowing Ability (MASA) having 94% sensitivity and 66% specificity, and the Water Swallow Test (WST) having 85% sensitivity and 75% specificity in acute stroke patients, highlighting the need to select appropriate tools for accurate dysphagia diagnosis and management (14, 16, 17). And problems such as over-prediction or under-prediction often exist. Moreover, due to varying pathologies (neurogenic vs. myogenic dysphagia), the effectiveness of these scales can differ (18, 19). There is a lack of comprehensive reviews on the effectiveness of non-instrumental bedside scales specifically for stroke patients. Therefore, our systematic review aimed to determine which non-instrumental assessment methods (clinical or comprehensive) are currently used to screen for dysphagia in stroke and which tool is more accurate for dysphagia screening in stroke patients after gold marker validation.

## Methods

This systematic review was registered on the PROSPERO platform with the registration number CRD42023494692 (20). The network meta-analysis (NMA) was conducted and reported following the Preferred Reporting Items for Systematic Reviews and Meta-Analyses (PRISMA) 2020 statement and checklist (21). The PRISMA statement and checklist (Supplementary Files S1, S2) are designed to enhance the transparency and necessity of reporting in systematic reviews.

### Eligibility criteria

Inclusion criteria: Studies were included in this systematic review if they met the following criteria: (1) patients diagnosed with stroke by imaging methods such as CTA or MRI; (2) studies utilized bedside screening tools to assess dysphagia; (3) sensitivity and specificity were used as primary outcome measures; (4) studies included patients in the acute phase of stroke; (5) There was sufficient information in English to demonstrate that they described a comprehensive nursing or MDT swallowing assessment to screen for dysphagia in stroke patients.

Studies were excluded if: (1) The study population included non-stroke patients; (2) Outcome indicators reported did not directly diagnose dysphagia; (3) There was no standard comparison to assess the accuracy of the results; (4) Reviews, conference abstracts, commentaries, open letters, editorials, and errata were excluded due to their inherent brevity.

### Data sources and search strategies

For data sources and search strategies, a series of relevant keywords were planned based on insights from evidence-based medicine experts. Studies included in this meta-analysis were diagnostic accuracy studies, randomized controlled trials, and prospective cohort studies that reported on the effectiveness or accuracy of dysphagia screening tools in stroke patients. Systematic searches were conducted in typical databases: Embase, PubMed, Web of Science, Scopus, and CINAHL. All publications up to 10 December 2023, were included. Terms related to dysphagia, assessment tools, and accuracy, including MeSH terms and free-text terms, were used to retrieve all relevant literature. The search strategies used in this review are outlined in Supplementary Table S1, summarizing the retrieval information for each database.

### Study selection

The study selection and review process was independently conducted by two reviewers, YJ and YC. The initial steps included eliminating duplicates and reading titles to exclude reviews, conference abstracts, letters, protocols, narrative reviews, and editorials. The remaining records underwent preliminary screening based on titles and abstracts, followed by a comprehensive evaluation through full-text reading. Any differences between the two primary reviewers were resolved through discussion. If unresolved differences persisted, a third reviewer (HJ) was consulted for resolution.

### Data extraction

For data extraction from eligible studies, two reviewers (SH and DZ) independently carried out this process using a predefined form. The following detailed information was captured: (1) Author names; (2) Publication year; (3) Demographic characteristics of the study population (age, gender, cohort); (4) Sample size recruited and ultimately included in the analysis; (5) Number of negative and positive samples; (6) Evaluation methods used for comparison; (7) Scales used; (8) Related indices of scale accuracy. In cases where the two primary reviewers had disagreements about data extraction, a third reviewer (YL) arbitrated and made the final decision.

### Quality assessment

The Quality Assessment of Diagnostic Accuracy Studies 2 (QUADAS-2) tool was utilized for evaluating the quality of each study. Two teams independently assessed the quality: Team 1 comprised YJ and YL, while Team 2 consisted of SH and RP. Disagreements were resolved by a third party (HJ). The evaluation was based on four parts: patient selection, index test, reference standard, and flow and timing. QUADAS-2 consists of 14 items, each rated as “yes” for meeting standards, “no” for not meeting or unmentioned standards, and “unclear” for insufficient information. Studies were finally classified as high, medium, or low quality. Publication bias was examined using funnel plots and Egger’s regression test, with *p* < 0.10 indicating evidence of publication bias (Supplementary Table S2).

### Conceptual mapping of measures

Statistical analysis was conducted using R version 4.3.2. Meta and meta for packages were utilized to aggregate sensitivity, specificity, positive predictive value, and negative predictive value as effect size statistics, each with their 95% confidence intervals (CIs). Considering the study’s focus on evaluating various screening tools based on original research, a consistency model was used for NMA and result ranking. Network package and related commands processed data, producing network relationship diagrams, forest plots, radar charts, and funnel plots. Funnel plots were used to identify publication bias, while predictive interval plots (95% CIs and 95% PrIs) assessed the heterogeneity of the combined results. Forest plots and radar charts presented the likelihood of each screening tool being the best choice.

## Results

### Search results

**Figure 1:**
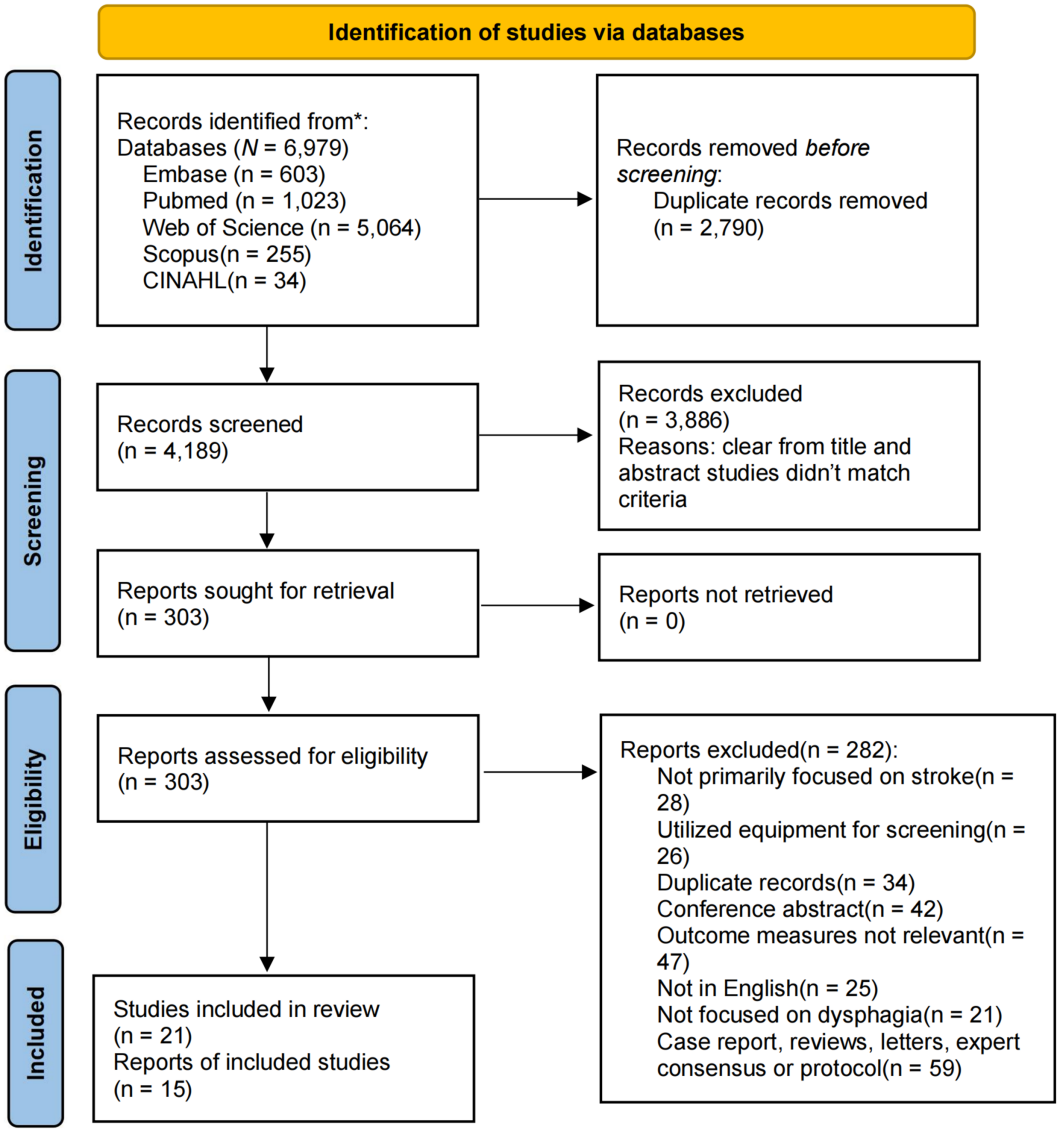
Flowchart of the study selection.

Our study involved a comprehensive literature search across five independent electronic databases, resulting in the retrieval of 6,979 records. These databases included Embase, PubMed, Web of Science, CINAHL, and Scopus. After eliminating 2,790 duplicates, 4,189 papers remained. Through title and abstract screening, 3,886 studies were excluded. Further full-text review led to the exclusion of an additional 282 studies. Consequently, 21 studies met the inclusion criteria and were incorporated into the systematic review, and 15 were included in the quantitative analysis (11, 12, 14, 15, 22–38) (Figure 1).

### Study characteristics

This NMA included 3,314 participants from 10 countries, comprising various types of stroke patients with and without dysphagia. The analysis evaluated the accuracy of 6 assessment tools across 21 studies (11, 12, 14, 15, 22–38). There are 15 studies utilized VFSS or FEES as gold standards for sensitivity and specificity (11, 12, 22, 24, 27–31, 33–38). MASA and SLP were used as comparative baselines in some studies (14, 15, 23, 25, 26, 32). The research involved both cross-sectional and case–control studies, including a multi-center observational study (11, 12, 14, 15, 22–38). Sensitivity and specificity of the various assessment tools ranged from 0.21 to 1.00 and 0.41 to 0.98, respectively. Detailed information about the studies included in the NMA can be found in Table 1.

**Table 1:** Summary of non-instrumental screening and assessment tools for post-stroke dysphagia.

Study	Country	Study design	Population	Sample size	Sample analyzed	Age (years)	Male *n*, (%)	Reference test	Test
Pacheco Castilho 2020	Brazil	Cross sectional	Stroke	60	60	64.9	33 (55.0)	VFSS	BSST
Umay 2018	Turkey	Cross sectional	Hemispheric stroke	128	113	66.71	61 (54.0)	FEES	GUSS
Simpelaere 2023	Belgium	Retrospective observational study	Acute stroke	115	115	82	66 (57.4)	SLP	MASA
Perry 2001	UK	Cross sectional	Stroke	200	200	71.6	111 (55.5)	SLP	WST
Umay 2018	Turkey	Cross sectional	Acute stroke	174	141	63.27	94 (66.7)	FEES	MASA
Immovilli 2020	Italy	Prospective observational study	Acute Stroke	120	120	67.4	67 (55.8)	FEES	BSST
Gandolfo 2019	Italy	Multicenter observational study	Stroke	249	249	72.6	126 (50.6)	SLP	WST
Sherman 2018	Canada	Retrospective observational study	Ischemic stroke	221	147	68	82 (56.0)	VFSS	DTNA
Warnecke 2017	Korea	Cross sectional	Acute stroke	121	100	73.6	56 (56.0)	FEES	GUSS
Behera 2018	US	prospective observational study	Stroke	225	225	68.4	122 (54.2)	MASA	WST
Edmiaston 2014	US	Cross sectional	Acute stroke	225	225	63	114 (50.9)	VFSS	WST
Somasundaram 2014	Germany	Cross sectional	Left-Hemispheric Middle Cerebral Artery Stroke	67	67	68	45 (67.0)	FEES	WST
Schrock 2011	US	Cohort study	Acute Stroke	283	283	65	144 (51.0)	VFSS	DTNA
Kopey 2011	US	Cross sectional	Acute stroke	350	223	62.5	107 (48.0)	VFSS	WST
Antonios 2010	US	Cross sectional	Acute stroke	150	150	64.5	70 (46.7)	VFSS	MASA
Bravata 2009	US	Retrospective observational study	Acute ischemic stroke	101	101	64.8	67 (66.3)	SLP	DTNA
Martino 2008	Canada	Cross sectional	Stroke	311	311	69.2	183 (58.8)	VFSS	BSST
Cummings 2015	US	Cross sectional	Stroke	49	49	71.7	25	SLP	DTNA
Benfield 2021	UK	Prospective observational study	Acute stroke	47	47	73	24(51.1)	VFSS	DTNA
Toscano 2018	UK	Prospective observational study	Stroke	52	50	74	33(63.5)	FEES	BSST
Rofes 2014	Spain	Retrospective observational study	Stroke	66	66	73.5	37 (56.1)	VFSS	V-VST

BSST, Bedside Swallow Screening Tool; WST, Water Swallow Test; DTNA, Dysphagia Trained Nurse Assessment.

### Study quality

The risk of bias assessment of the studies included in this NMA showed that 7 studies were of high quality and 7 studies were of moderate quality (11, 12, 22, 23, 25–28, 31, 33, 35–38). Most studies had a low risk of bias, with 15 studies enrolling consecutive cases and 19 studies avoiding a case–control design (11, 12, 14, 15, 22, 24–35, 37, 38). Most studies managed to exclude unsuitable patients, interpret test results without knowledge of the reference standard, set predefined thresholds, use the same reference standard for patients, and analyze all included patients. A detailed description of the population, age, and sample size was provided (Supplementary Table S2). The funnel plots for sensitivity, specificity, and accuracy exhibit a symmetrical distribution around the mean effect size, indicating minimal publication bias across the included studies. Slight skewness observed in the specificity plot suggests minor bias, but overall, the symmetry in the data supports the robustness of the meta-analysis findings (Supplementary Figures S1–S3).

### Meta-analysis

**Figure 2:**
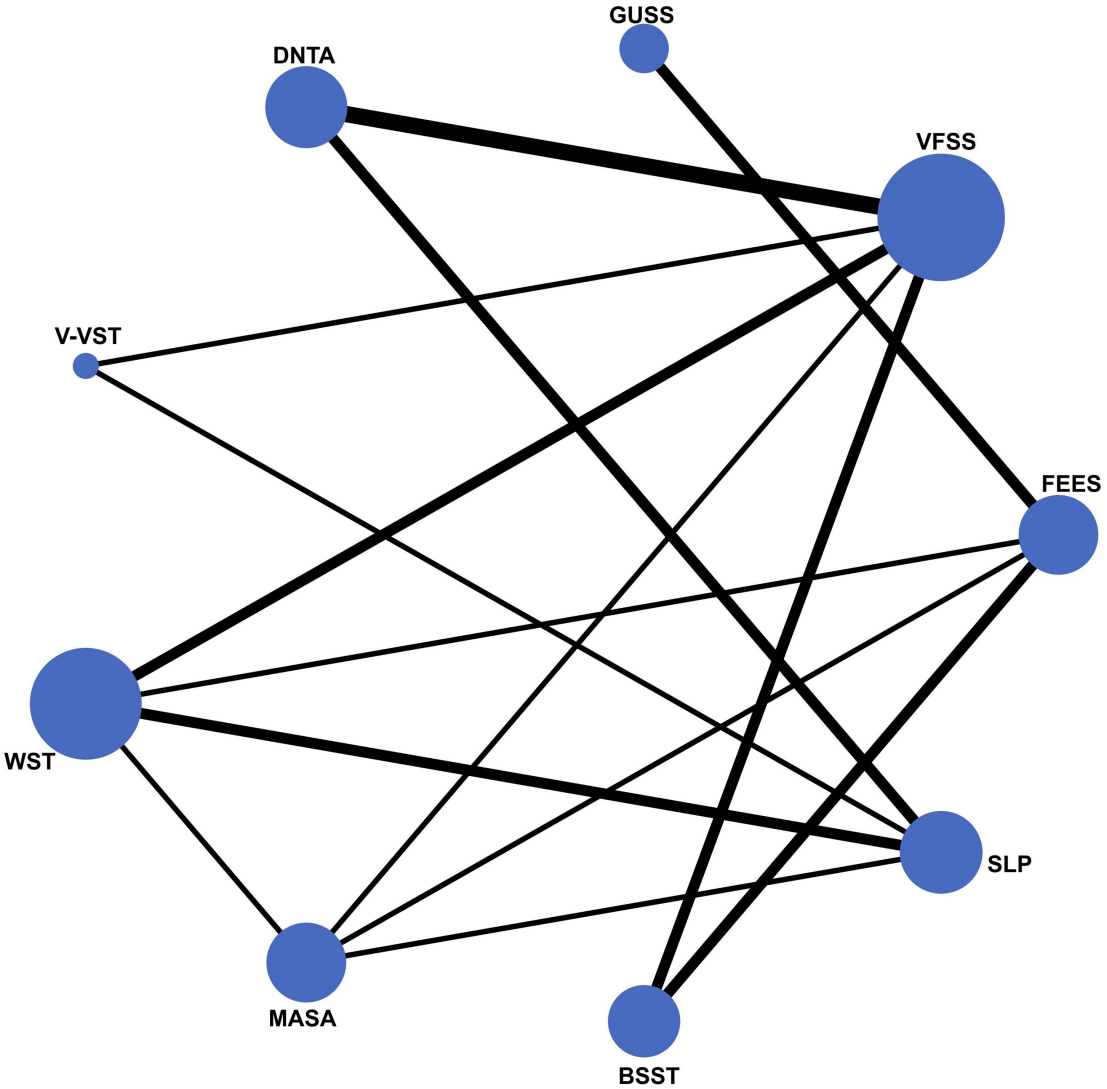
Network meta-analysis graph of comparative effectiveness among different screening tools.

The network diagram in this study illustrates the inclusion of nine dysphagia assessment tools, with FEES and VFSS prominently positioned as the gold standards, indicated by their central and largest nodes in the network. This prominence reflects their extensive use and frequent comparison with other assessment tools. Conversely, tools such as MASA, V-VST, and GUSS are represented by smaller nodes, suggesting that fewer studies have compared these tools directly with the gold standards. This network visualization underscores the central role of FEES and VFSS in dysphagia screening while highlighting the relative under representation of other tools in the literature (Figure 2).

### Heterogeneity test

**Figure 3:**
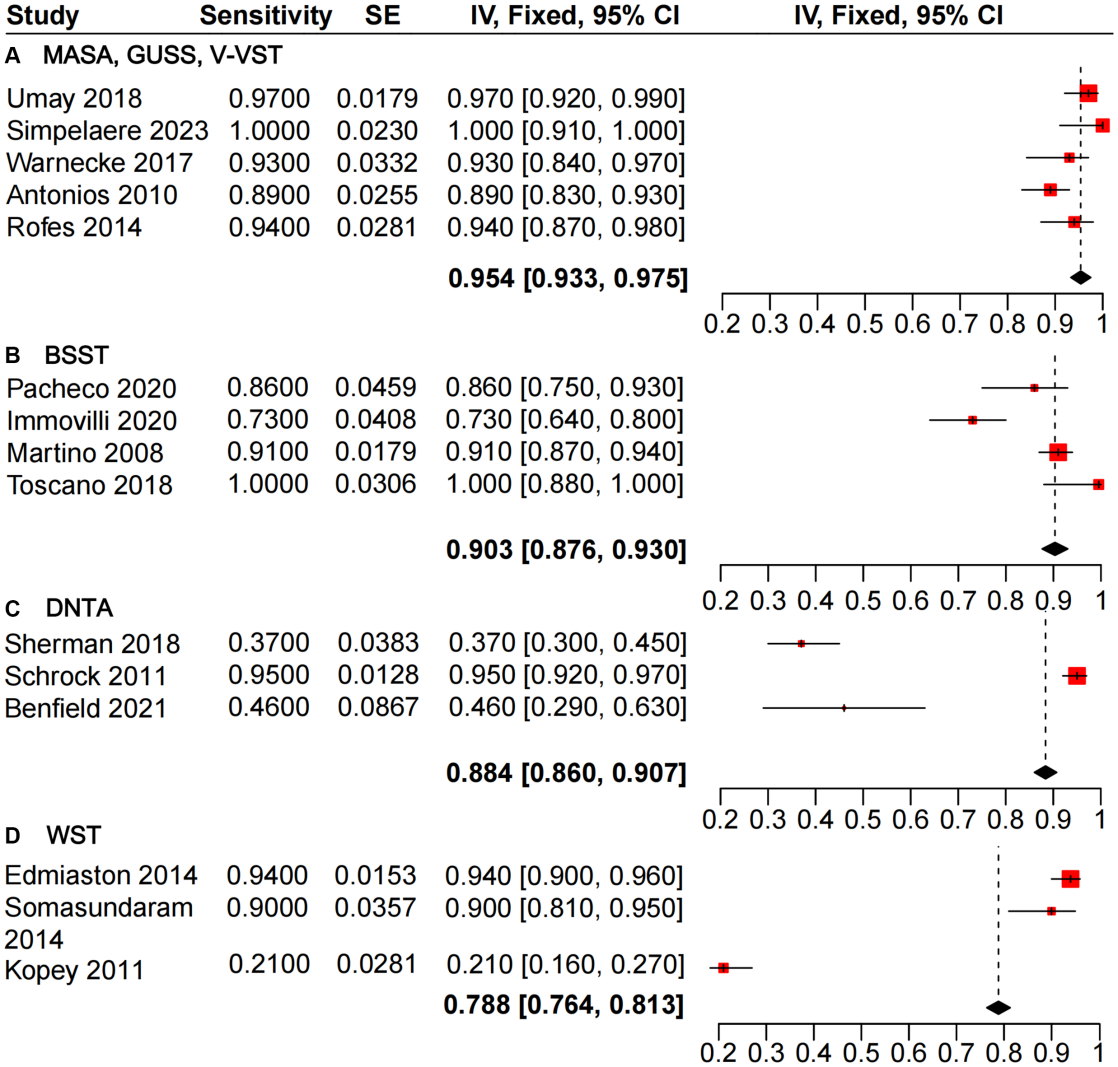
Forest plot of sensitivity analysis for non-instrumental dysphagia screening tools.

**Figure 4:**
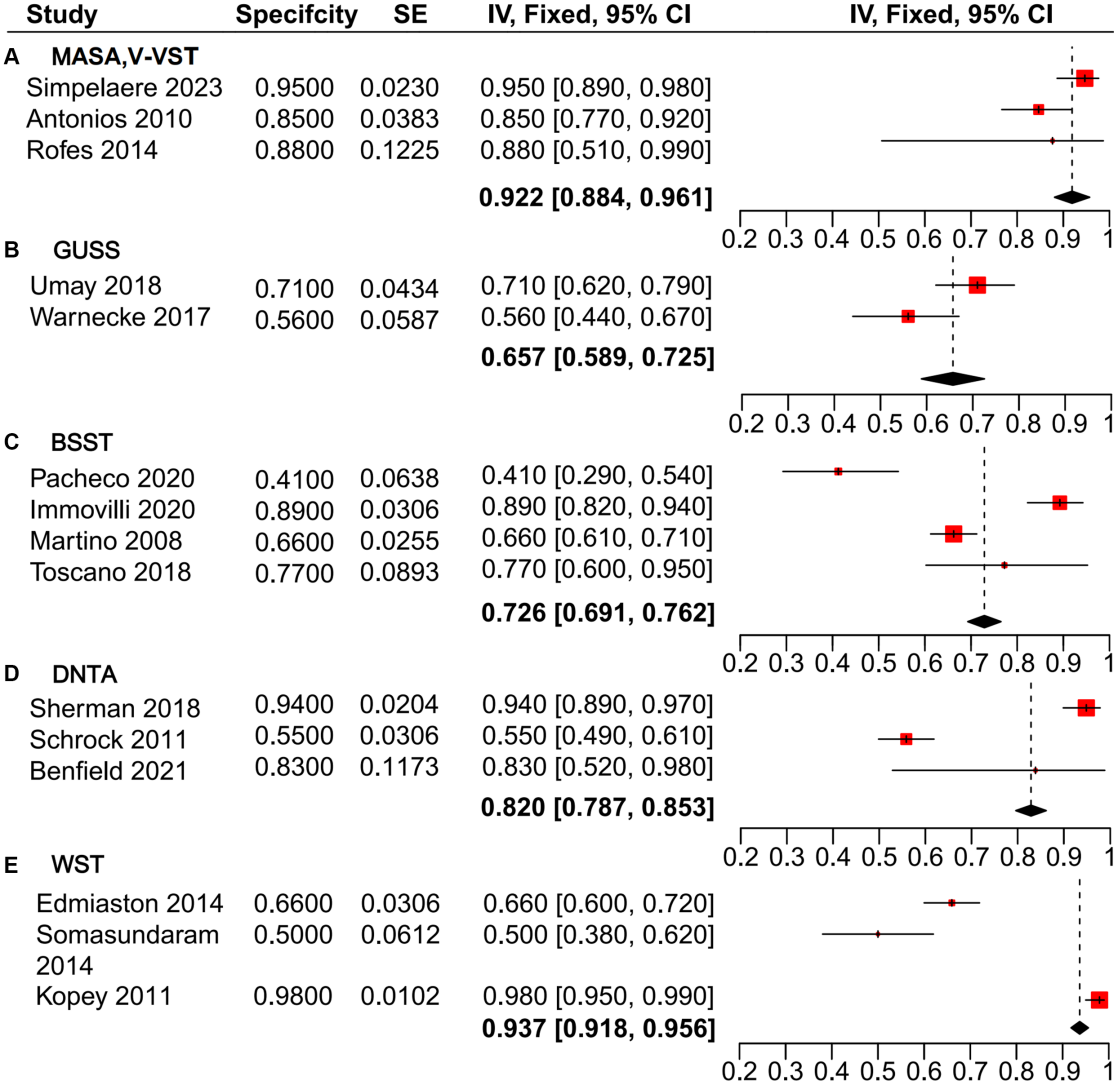
Forest plot of specificity analysis for non-instrumental dysphagia screening tools.

The heterogeneity analysis of sensitivity for the GUSS, V-VST, and MASA dysphagia screening tools revealed minimal variability across the five studies evaluated. These tools demonstrated high sensitivity, with a pooled estimate from the mixed-effects model of 0.954 [0.933, 0.975], outperforming BSST (0.903 [0.876, 0.930]), DTNA (0.884 [0.860, 0.907]), and WST (0.788 [0.764, 0.813]) (Figure 3). Similarly, the heterogeneity analysis of specificity (Figure 4) indicated that MASA and V-VST, along with GUSS, exhibited low heterogeneity. MASA and V-VST, in particular, showed more stable and higher specificity, with a pooled estimate from the mixed-effects model of 0.922 [0.884, 0.961]. These findings highlight the robustness and reliability of MASA and V-VST as superior tools for dysphagia screening in stroke patients, offering consistent performance across diverse study settings.

### Diagnostic accuracy

**Figure 5:**
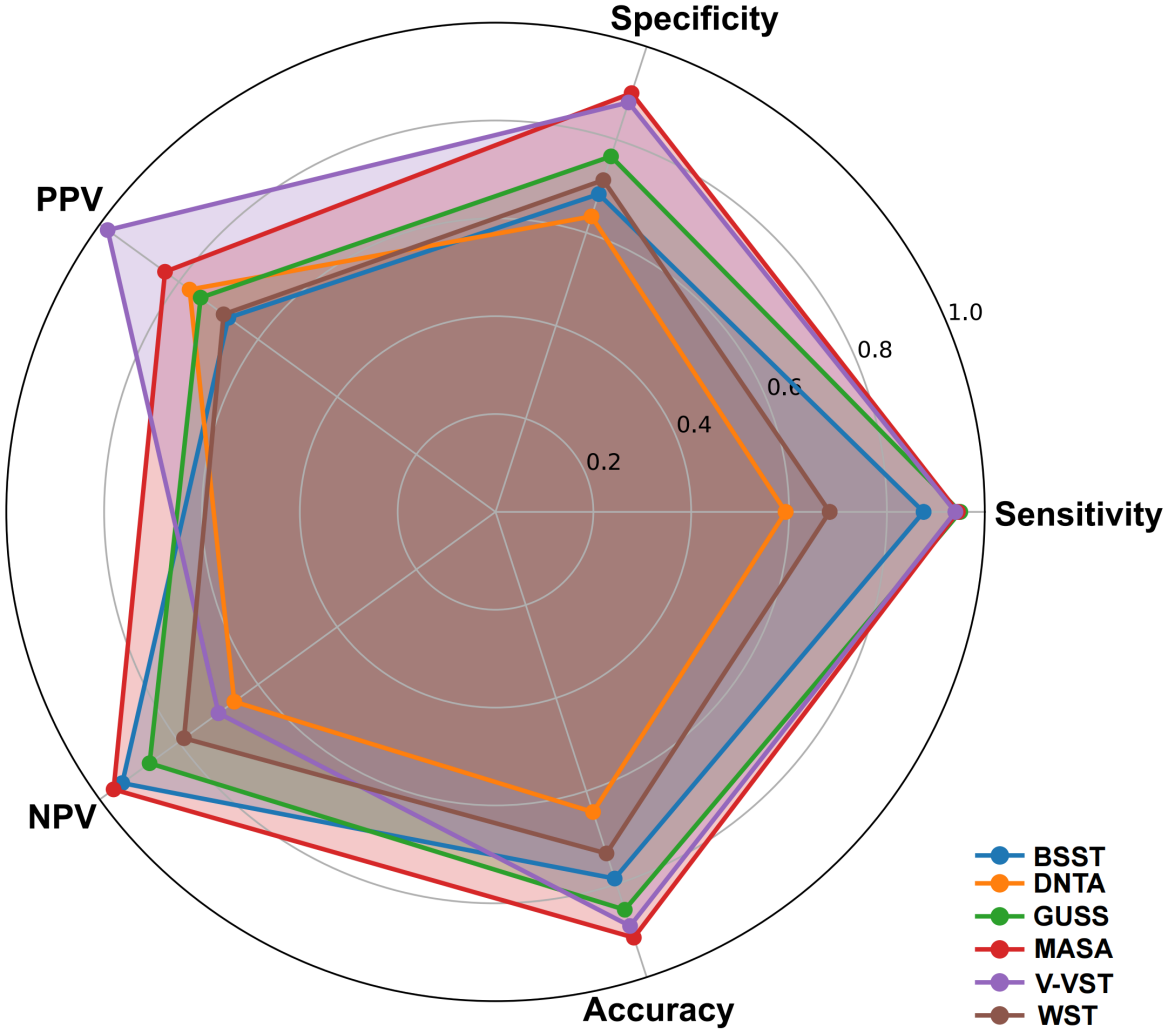
Radar chart of performance metrics for non-instrumental dysphagia screening tools using gold standard benchmark.

The radar chart delineates the performance metrics of various non-instrumental dysphagia assessment tools, bench marked against the gold-standard diagnostic methods VFSS and FEES. GUSS, MASA, and V-VST exhibit superior performance across key parameters, including sensitivity, specificity, positive predictive value (PPV), negative predictive value (NPV), and overall accuracy. GUSS and MASA demonstrate particularly high sensitivity and specificity, underscoring their reliability in detecting and excluding dysphagia. Similarly, V-VST shows robust performance with high specificity and sensitivity, complemented by strong PPV and NPV values. Conversely, WST, while maintaining good sensitivity, shows lower specificity, indicating a tendency for over-prediction. BSST and BTNA display comparatively lower sensitivity and specificity, suggesting lesser reliability. Overall, GUSS, MASA, and V-VST align closely with gold-standard benchmarks, highlighting their efficacy and reliability in clinical dysphagia screening for stroke patients (Figure 5; Supplementary Table S3).

## Discussion

Accurate screening for dysphagia is crucial in mitigating risks such as aspiration pneumonia and malnutrition (38). This network meta-analysis provides a comprehensive evaluation of various non-instrumental dysphagia screening tools against gold-standard methods (VFSS and FEES) for stroke patients. The findings highlight the efficacy and reliability of these tools, with implications for clinical practice. Furthermore, in this review, the smaller heterogeneity observed in the evaluation effects of some screening tools through subgroup analysis of the included studies indicated that the results were consistent in different settings, while the symmetrical distribution in the funnel plot indicated minimal publication bias, which enhanced the credibility of our meta-analysis.

The results of this study highlight the robustness and reliability of GUSS, MASA, and V-VST as primary tools for dysphagia screening. Their consistent performance across different studies underscores their potential for widespread clinical application. This robustness is particularly important in diverse clinical settings, ensuring accurate patient assessments regardless of specific conditions. MASA and V-VST demonstrate the best overall performance for diagnosing dysphagia and screening healthy individuals, though further research is needed on V-VST’s comparison to gold standards to support its clinical adoption. Additionally, while GUSS shows high sensitivity, its lower specificity may limit its use in excluding non-dysphagic patients. However, GUSS remains a viable option for early dysphagia screening in acute stroke patients.

MASA, known for its high specificity in dysphagia screening among stroke patients, incorporates 24 comprehensive clinical items (39). These items evaluate oral motor skills, pharyngeal reflexes, laryngeal movement, and the coordination of chewing and swallowing. A score above 178 on MASA suggests the absence of dysphagia. Validated through techniques such as FEES, MASA has demonstrated robust sensitivity and specificity (29). It has been extensively studied in acute stroke settings across diverse regions including the U.S., Belgium, and Turkey, which supports its application in these contexts (14, 22, 32). The high specificity of MASA is significant in clinical practice, particularly in stroke rehabilitation, as it minimizes false-positive rates, thereby ensuring that patients who truly require intervention for dysphagia are accurately identified and managed. Moreover, studies like that by Tomoya Omura (40) report MASA’s effectiveness in aspiration detection, potentially aiding in the prevention of aspiration pneumonia. However, its comprehensive nature makes it detailed and time-consuming, posing challenges in fast-paced clinical settings. Professional training, typically provided by speech pathologists, is essential for accurate administration and interpretation of MASA, which may limit its accessibility in general clinical practice (22). MASA’s scope of application is expanding beyond stroke patient assessments to include evaluating the effects of treatments like percutaneous auricular vagus nerve stimulation therapy, as studied by Wang et al., and assessing swallowing abilities in sarcopenia patients (22). Integrating this training into clinical practice, especially in busy or resource-limited settings, is challenging due to MASA’s detailed nature and the time investment required for its administration. These factors can limit MASA’s practicality in some healthcare environments, necessitating consideration of the healthcare setting’s capacity when choosing to implement MASA for dysphagia assessment.

Comparative analysis reveals that while tools like BSST and WST are useful, they exhibit lower diagnostic accuracy compared to GUSS, MASA, and V-VST. This has practical implications, especially in resource-limited settings where selecting the most reliable tool is critical for accurate patient assessments and avoiding unnecessary interventions. The superior performance of GUSS, MASA, and V-VST suggests they should be preferred in clinical practice, particularly where precise diagnosis can significantly impact patient management and outcomes. In contrast, tools like BSST, WST, and DNTA show inconsistent results across different versions and upgraded evaluation tools for dysphagia screening. For example, Edmiaston and Somasundaram (27, 36) reported WST sensitivity above 0.9 but lower specificity, whereas Kopey (30), using the gold-standard comparison, reported completely opposite findings. This discrepancy may be attributed to Edmiaston and Somasundaram’s use of more controlled and standardized procedures, enhancing the reliability of dysphagia detection (27, 36). In contrast, Kopey’s use of the less standardized 3-sip test resulted in higher variability and lower sensitivity (30). Additionally, differences in the administrators and managers of WST validation studies and the diverse patient populations included in these studies could contribute to the significant variability in outcomes.

The meta-analysis indicates that BSST, with its high NPV and specificity, could have a unique role in assessing dysphagia in acute stroke patients, such as in assessing swallowing function during rehabilitation. The simplicity and rapid application of BSST make it a practical, non-invasive option during acute hospital admissions (28). However, its effectiveness heavily relies on the practitioner’s experience and thorough assessment skills (31). Therefore, standardized training and clear procedural guidelines are essential to maximize its potential. BSST involves a comprehensive set of assessments, including recording patient characteristics, evaluating speech and communication skills, conducting facial and oral motor examinations, monitoring oxygen saturation, performing water swallow tests, and using thickened liquids for evaluation (31). The dependence on clinical experience and detailed evaluation of patient factors, such as alertness, language ability, facial symmetry, and apraxia, means BSST lacks uniform content, affecting its reproducibility and practicality across different clinical settings (11). Similarly, DNTA’s consistency is compromised due to variability in trained nurses and differing assessment practices. Overall, the heterogeneity in the screening effectiveness of BSST and DNTA is high, likely due to the lack of consistent assessment protocols and the use of varied innovative approaches by different researchers. Our meta-analysis showed that the overall performance of BSST and DNTA was inferior to GUSS, MASA, and V-VST, which may limit the future applicability of BSST and DNTA in research and clinical practice.

Our network meta-analysis underscores the importance of personalized dysphagia screening tailored to patient conditions and stroke stages. Tools like the GUSS, V-VST, MASA and WST offer unique advantages, particularly for acute stroke patients. WST is favored for its simplicity and reliability in initial screenings, while MASA, known for its high accuracy, is more complex and time-consuming, necessitating selective use. A patient-centered approach is essential, with healthcare professionals evaluating each patient’s condition, aspiration risk, and cooperation level to ensure accurate diagnosis and patient comfort, thereby enhancing care quality. Integrating tools, such as combining GUSS’s high sensitivity with WST’s high specificity, can significantly improve diagnostic accuracy, particularly in preventing and managing post-stroke dysphagia and aspiration. However, GUSS and V-VST, despite their safety benefits, can be cumbersome and time-consuming. Clinical assessments often rely on a single scale, each with its strengths and limitations. While combining multiple scales holds promise for improved accuracy, the economic and social impacts of this approach require further exploration. By considering patient conditions and aspiration risks, and integrating diverse tools, healthcare professionals can reduce misdiagnosis risks and provide a more effective foundation for managing post-stroke dysphagia.

Despite the comprehensive nature of this network meta-analysis, several limitations should be acknowledged. First, our study focused on dysphagia screening tools, excluding studies that specifically validated scales with aspiration as the primary patient outcome. This exclusion might have limited our understanding of tools specifically designed to prevent aspiration pneumonia. Future research should address limitations such as the exclusion of aspiration-specific outcomes and study heterogeneity. Second, since many of the original studies included did not specify whether they included patients with language disorders, this may increase heterogeneity and may lead to some differences in our findings. Although we accounted for these differences through statistical methods, minor inconsistencies may still exist. Lastly, while we aimed to provide a thorough analysis, the reliance on published data means that potential publication bias and incomplete reporting could affect the robustness of our conclusions. Future research should aim to address these limitations by including more diverse patient outcomes and ensuring consistency in study designs and reporting standards.

## Conclusion

This network meta-analysis highlights the importance of accurate dysphagia screening tools for stroke patients. GUSS, MASA, and V-VST emerged as the most reliable, demonstrating superior sensitivity and specificity, suitable for diverse clinical settings. While BSST and WST have practical uses, their lower diagnostic accuracy suggests a more selective application. The findings emphasize the need for personalized screening approaches tailored to individual patient conditions and stroke stages. By broadening patient outcomes and standardizing methodologies, future studies can improve the effectiveness of dysphagia screening tools, enhancing patient care and reducing complications.

### What is already known on this topic

Dysphagia is a prevalent and serious complication in stroke patients, necessitating early and accurate screening to improve outcomes.

Existing dysphagia screening tools exhibit varied efficacy across different patient populations; however, this study identifies MASA, GUSS, and V-VST as reliable and relatively accurate tools for dysphagia screening.

There is a critical need for personalized screening approaches in dysphagia management post-stroke, highlighting the gap in comprehensive comparative analyses.

### What this study adds

- This study provides a direct comparison of 6 non-instrumental bedside dysphagia screening tools, revealing specific strengths and weaknesses in acute stroke settings.
- Our findings quantify the sensitivity and specificity of each tool, offering concrete data to guide clinicians in selecting the most effective screening method for stroke-induced dysphagia.
- The analysis underscores the importance of patient-specific factors and stroke stages in choosing dysphagia screening tools, advancing personalized care approaches.

### How this study might affect research, practice, or policy

- This study’s insights can inform the development of tailored dysphagia screening protocols, potentially leading to revised clinical guidelines that enhance patient care and outcomes.
- The comparative effectiveness data provided may drive future research toward innovative screening tools and methodologies, particularly those incorporating patient-specific variables and stroke recovery stages.

## Data availability statement

The original contributions presented in the study are included in the article/Supplementary material, further inquiries can be directed to the corresponding authors.

## Author contributions

YJ: Writing – review & editing, Writing – original draft, Software, Methodology. YC: Writing – original draft, Formal analysis, Data curation. RP: Writing – review & editing, Conceptualization. DZ: Writing – original draft, Data curation. SH: Writing – original draft, Data curation. HJ: Writing – original draft, Supervision, Project administration, Funding acquisition. YL: Writing – original draft, Supervision, Project administration.
