# Supplementary material for: Advancing stroke patient care: a network meta-analysis of dysphagia screening efficacy and personalization
Source: Front Neurol. 2024 Aug 6;15:1380287. doi: 10.3389/fneur.2024.1380287 (PMC11333969; doi:10.3389/fneur.2024.1380287)
Supplement: Supplementary file 3 [file Table_1.DOCX]

**Supplementary materials**

**Table S1. Search strategies**

| Keywords | Search strategy | Results |
| --- | --- | --- |
| **Pubmed** |  |  |
| #1 Deglutition Disorders | "deglutition disorders"[MeSH Terms] OR "deglutition disorders"[Title/Abstract] OR "disorders deglutition"[Title/Abstract] OR "Dysphagia"[Title/Abstract] OR "deglutition disorder"[Title/Abstract] OR "swallowing disorders"[Title/Abstract] OR "swallowing disorder"[Title/Abstract] OR "oropharyngeal dysphagia"[Title/Abstract] OR "dysphagia oropharyngeal"[Title/Abstract] OR "esophageal dysphagia"[Title/Abstract] OR "dysphagia esophageal"[Title/Abstract] OR "Deglutition"[MeSH Terms] OR "Deglutitions"[Title/Abstract] OR "Deglutition"[Title/Abstract] OR "Swallowing"[Title/Abstract] OR "Swallowings"[Title/Abstract] | 98,126 |
| #2 Sensitivity/ specificity | "predictive value of tests"[MeSH Terms] OR "predictive values of tests"[Title/Abstract] OR "predictive value of test"[Title/Abstract] OR "negative predictive value"[Title/Abstract] OR "negative predictive values"[Title/Abstract] OR "sensitivity and specificity"[MeSH Terms] OR "predictive value of tests"[Title/Abstract] OR ("Specificity"[Title/Abstract] AND "Sensitivity"[Title/Abstract]) OR ("Sensitivity"[Title/Abstract] AND "Specificity"[Title/Abstract]) OR "Sensitivity"[Title/Abstract] OR "Specificity"[Title/Abstract] OR "Diagnosis"[Title/Abstract] OR "Diagnoses"[Title/Abstract] OR ("Diagnoses"[Title/Abstract] AND "Examinations"[Title/Abstract]) OR ("Examination"[Title/Abstract] AND "Diagnoses"[Title/Abstract]) OR "antemortem diagnosis"[Title/Abstract] OR "postmortem diagnosis"[Title/Abstract] OR "Validity"[Title/Abstract] OR "Accuracy"[Title/Abstract] OR "diagnosis, differential"[MeSH Terms] OR "diagnosis differential"[Title/Abstract] OR "diagnoses differential"[Title/Abstract] OR "differential diagnoses"[Title/Abstract] OR "diagnostic errors"[MeSH Terms] OR "diagnostic errors"[Title/Abstract] OR "diagnostic error"[Title/Abstract] OR "diagnostic blind spots"[Title/Abstract] OR "Misdiagnoses"[Title/Abstract] | [4,369,054](https://pubmed.ncbi.nlm.nih.gov/?term=("predictive+value+of+tests"[MeSH+Terms]+OR+"predictive+values+of+tests"[Title/Abstract]+OR+"predictive+value+of+test"[Title/Abstract]+OR+"negative+predictive+value"[Title/Abstract]+OR+"negative+predictive+values"[Title/Abstract]+OR+"sensitivity+and+specificity"[MeSH+Terms]+OR+"predictive+value+of+tests"[Title/Abstract]+OR+("Specificity"[Title/Abstract]+AND+"Sensitivity"[Title/Abstract])+OR+("Sensitivity"[Title/Abstract]+AND+"Specificity"[Title/Abstract])+OR+"Sensitivity"[Title/Abstract]+OR+"Specificity"[Title/Abstract]+OR+"Diagnosis"[Title/Abstract]+OR+"Diagnoses"[Title/Abstract]+OR+("Diagnoses"[Title/Abstract]+AND+"Examinations"[Title/Abstract])+OR+("Examination"[Title/Abstract]+AND+"Diagnoses"[Title/Abstract])+OR+"antemortem+diagnosis"[Title/Abstract]+OR+"postmortem+diagnosis"[Title/Abstract]+OR+"Validity"[Title/Abstract]+OR+"Accuracy"[Title/Abstract]+OR+"diagnosis,+differential"[MeSH+Terms]+OR+"diagnosis+differential"[Title/Abstract]+OR+"diagnoses+differential"[Title/Abstract]+OR+"differential+diagnoses"[Title/Abstract]+OR+"diagnostic+errors"[MeSH+Terms]+OR+"diagnostic+errors"[Title/Abstract]+OR+"diagnostic+error"[Title/Abstract]+OR+"diagnostic+blind+spots"[Title/Abstract]+OR+"Misdiagnoses"[Title/Abstract])+AND+(("1900/01/01"[Date+-+Publication]+:+"2023/12/09"[Date+-+Publication]))&ac=no&sort=relevance) |
| #3 Scale | "Scale"[Title/Abstract] OR "Scale"[Title/Abstract] OR "tool"[Title/Abstract] OR "assessment"[Title/Abstract] OR "test"[Title/Abstract] OR "examination"[Title/Abstract] OR "screen"[Title/Abstract] OR "Instrument"[Title/Abstract] OR "Questionnaire"[Title/Abstract] OR "Index"[Title/Abstract] OR "Battery"[Title/Abstract] OR "Checklist"[Title/Abstract] OR "Survey"[Title/Abstract] OR "Profile"[Title/Abstract] OR "Evaluation"[Title/Abstract] OR "Appraisal"[Title/Abstract] OR "Measurement"[Title/Abstract] OR "Rating"[Title/Abstract] OR "Metrics"[Title/Abstract] OR "Validation"[Title/Abstract] OR "Technique"[Title/Abstract] OR "Method"[Title/Abstract] OR "Approach"[Title/Abstract] | 11,304,981 |
| #4 Stroke | "Stroke"[MeSH Terms] OR "Stroke"[Title/Abstract] OR "Strokes"[Title/Abstract] OR "cerebrovascular accident"[Title/Abstract] OR "cerebrovascular accidents"[Title/Abstract] OR "cerebral stroke"[Title/Abstract] OR "cerebral strokes"[Title/Abstract] OR "stroke cerebral"[Title/Abstract] OR "strokes cerebral"[Title/Abstract] OR "cerebrovascular apoplexy"[Title/Abstract] OR "apoplexy cerebrovascular"[Title/Abstract] OR "brain vascular accident"[Title/Abstract] OR (("blood vessels"[MeSH Terms] OR ("blood"[All Fields] AND "vessels"[All Fields]) OR "blood vessels"[All Fields] OR "Vascular"[All Fields] OR "neovascularization, pathologic"[MeSH Terms] OR ("neovascularization"[All Fields] AND "pathologic"[All Fields]) OR "pathologic neovascularization"[All Fields] OR "vascularisation"[All Fields] OR "vascularization"[All Fields] OR "vascularisations"[All Fields] OR "vascularise"[All Fields] OR "vascularised"[All Fields] OR "vascularities"[All Fields] OR "vascularitis"[All Fields] OR "vascularity"[All Fields] OR "vascularizations"[All Fields] OR "vascularize"[All Fields] OR "vascularized"[All Fields] OR "vascularizes"[All Fields] OR "vascularizing"[All Fields] OR "vasculars"[All Fields]) AND "accidents brain"[Title/Abstract]) OR "cerebrovascular strokes"[Title/Abstract] OR "strokes cerebrovascular"[Title/Abstract] OR "stroke cerebrovascular"[Title/Abstract] OR "acute stroke"[Title/Abstract] OR "stroke acute"[Title/Abstract] | 372,731 |
|  | #1 and #2 and #3 and #4 | 603 |

**Table S1. Search strategies (continues)**

| Keywords | Search strategy | Results |
| --- | --- | --- |
| **Web of science** |  |  |
| #1 Deglutition Disorders | TS=("deglutition disorders" OR "disorders deglutition" OR "Dysphagia" OR "deglutition disorder" OR "swallowing disorders" OR "swallowing disorder" OR "oropharyngeal dysphagia" OR "dysphagia oropharyngeal" OR "esophageal dysphagia" OR "dysphagia esophageal" OR "Deglutition" OR "Deglutitions" OR "Swallowing" OR "Swallowings") | 53,099 |
| #2 Sensitivity/ specificity | TS=("predictive value of tests" OR "predictive values of tests" OR "predictive value of test" OR "negative predictive value" OR "negative predictive values" OR "sensitivity and specificity" OR ("Specificity" AND "Sensitivity") OR ("Sensitivity" AND "Specificity") OR "Sensitivity" OR "Specificity" OR "Diagnosis" OR "Diagnoses" OR ("Diagnoses" AND "Examinations") OR ("Examination" AND "Diagnoses") OR "antemortem diagnosis" OR "postmortem diagnosis" OR "Validity" OR "Accuracy" OR "diagnosis differential" OR "differential diagnosis" OR "differential diagnoses" OR "diagnostic errors" OR "diagnostic error" OR "diagnostic blind spots" OR "Misdiagnoses") | 6,061,140 |
| #3 Scale | TS=("Scale" OR "tool" OR "assessment" OR "test" OR "examination" OR "screen" OR "Instrument" OR "Questionnaire" OR "Index" OR "Battery" OR "Checklist" OR "Survey" OR "Profile" OR "Evaluation" OR "Appraisal" OR "Measurement" OR "Rating" OR "Metrics" OR "Validation" OR "Technique" OR "Method" OR "Approach") | 24,844,994 |
| #4 Stroke | TS=("Stroke" OR "Strokes" OR "cerebrovascular accident" OR "cerebrovascular accidents" OR "cerebral stroke" OR "cerebral strokes" OR "stroke cerebral" OR "strokes cerebral" OR "cerebrovascular apoplexy" OR "apoplexy cerebrovascular" OR "brain vascular accident" OR (("blood vessels" OR "Vascular" OR "neovascularization pathologic" OR "pathologic neovascularization" OR "vascularisation" OR "vascularization" OR "vascularisations" OR "vascularise" OR "vascularised" OR "vascularities" OR "vascularitis" OR "vascularity" OR "vascularizations" OR "vascularize" OR "vascularized" OR "vascularizes" OR "vascularizing" OR "vasculars") AND "accidents brain") OR "cerebrovascular strokes" OR "strokes cerebrovascular" OR "stroke cerebrovascular" OR "acute stroke" OR "stroke acute") | 466,141 |
|  | #1 and #2 and #3 and #4 | 1,023 |

**Table S1. Search strategies (continues)**

| **Keywords** | **Search strategy** | **Results** |
| --- | --- | --- |
| **Embase** |  |  |
| #1 Deglutition Disorders | 'deglutition disorder'/exp OR 'deglutition disorder' OR 'disorders deglutition' OR 'dysphagia'/exp OR 'dysphagia' OR 'deglutition disorder' OR 'swallowing disorder'/exp OR 'swallowing disorder' OR 'oropharyngeal dysphagia' OR 'dysphagia oropharyngeal' OR 'esophageal dysphagia' OR 'dysphagia esophageal' OR 'deglutition'/exp OR 'deglutition' OR 'deglutitions' OR 'swallowing' OR 'swallowings' | 141,435 |
| #2 Sensitivity/ specificity | ('predictive value'/exp OR 'predictive value of tests' OR 'predictive values of tests' OR 'predictive value of test' OR 'negative predictive value' OR 'negative predictive values' OR 'sensitivity and specificity'/exp OR 'sensitivity and specificity' OR ('specificity' AND 'sensitivity') OR ('sensitivity' AND 'specificity') OR 'sensitivity' OR 'specificity' OR 'diagnosis'/exp OR 'diagnosis' OR 'diagnoses' OR ('diagnoses' AND 'examinations') OR ('examination' AND 'diagnoses') OR 'antemortem diagnosis' OR 'postmortem diagnosis' OR 'validity' OR 'accuracy' OR 'differential diagnosis'/exp OR 'differential diagnosis' OR 'differential diagnoses' OR 'diagnostic errors'/exp OR 'diagnostic errors' OR 'diagnostic error' OR 'diagnostic blind spots' OR 'misdiagnoses') | 12,971,345 |
| #3 Scale | ('scale'/exp OR 'scale' OR 'tool' OR 'assessment' OR 'test' OR 'examination' OR 'screen' OR 'instrument' OR 'questionnaire'/exp OR 'questionnaire' OR 'index' OR 'battery' OR 'checklist' OR 'survey' OR 'profile' OR 'evaluation' OR 'appraisal' OR 'measurement' OR 'rating' OR 'metrics' OR 'validation' OR 'technique' OR 'method' OR 'approach') | 27,286,998 |
| #4 Stroke | ('stroke'/exp OR 'stroke' OR 'strokes' OR 'cerebrovascular accident' OR 'cerebrovascular accidents' OR 'cerebral stroke' OR 'cerebral strokes' OR 'stroke cerebral' OR 'strokes cerebral' OR 'cerebrovascular apoplexy' OR 'apoplexy cerebrovascular' OR 'brain vascular accident' OR (('blood vessel'/exp OR 'blood vessels' OR 'vascular' OR 'neovascularization, pathologic' OR 'pathologic neovascularization' OR 'vascularisation' OR 'vascularization' OR 'vascularisations' OR 'vascularise' OR 'vascularised' OR 'vascularities' OR 'vascularitis' OR 'vascularity' OR 'vascularizations' OR 'vascularize' OR 'vascularized' OR 'vascularizes' OR 'vascularizing' OR 'vasculars') AND 'accidents brain') OR 'cerebrovascular strokes' OR 'strokes cerebrovascular' OR 'stroke cerebrovascular' OR 'acute stroke' OR 'stroke acute') | 711,400 |
|  | #1 and #2 and #3 and #4 | 5,064 |

**Table S1. Search strategies (continues)**

| Keywords | Search strategy | Results |
| --- | --- | --- |
| **Scopus** |  |  |
| #1 Deglutition disorders | (TITLE-ABS-KEY("deglutition disorders") OR TITLE-ABS-KEY("disorders deglutition") OR TITLE-ABS-KEY("dysphagia") OR TITLE-ABS-KEY("deglutition disorder") OR TITLE-ABS-KEY("swallowing disorders") OR TITLE-ABS-KEY("swallowing disorder") OR TITLE-ABS-KEY("oropharyngeal dysphagia") OR TITLE-ABS-KEY("dysphagia oropharyngeal") OR TITLE-ABS-KEY("esophageal dysphagia") OR TITLE-ABS-KEY("dysphagia esophageal") OR TITLE-ABS-KEY("deglutition") OR TITLE-ABS-KEY("deglutitions") OR TITLE-ABS-KEY("swallowing") OR TITLE-ABS-KEY("swallowings")) | 115,761 |
| #2 Sensitivity/ specificity | (TITLE-ABS-KEY("predictive value of tests") OR TITLE-ABS-KEY("predictive values of tests") OR TITLE-ABS-KEY("predictive value of test") OR TITLE-ABS-KEY("negative predictive value") OR TITLE-ABS-KEY("negative predictive values") OR TITLE-ABS-KEY("sensitivity and specificity") OR (TITLE-ABS-KEY("specificity") AND TITLE-ABS-KEY("sensitivity")) OR TITLE-ABS-KEY("sensitivity") OR TITLE-ABS-KEY("specificity") OR TITLE-ABS-KEY("diagnosis") OR TITLE-ABS-KEY("diagnoses") OR (TITLE-ABS-KEY("diagnoses") AND TITLE-ABS-KEY("examinations")) OR (TITLE-ABS-KEY("examination") AND TITLE-ABS-KEY("diagnoses")) OR TITLE-ABS-KEY("antemortem diagnosis") OR TITLE-ABS-KEY("postmortem diagnosis") OR TITLE-ABS-KEY("validity") OR TITLE-ABS-KEY("accuracy") OR TITLE-ABS-KEY("differential diagnosis") OR TITLE-ABS-KEY("differential diagnoses") OR TITLE-ABS-KEY("diagnostic errors") OR TITLE-ABS-KEY("diagnostic error") OR TITLE-ABS-KEY("diagnostic blind spots") OR TITLE-ABS-KEY("misdiagnoses")) | 10,367,293 |
| #3 Scale | (TITLE-ABS-KEY("scale") OR TITLE-ABS-KEY("tool") OR TITLE-ABS-KEY("assessment") OR TITLE-ABS-KEY("test") OR TITLE-ABS-KEY("examination") OR TITLE-ABS-KEY("screen") OR TITLE-ABS-KEY("instrument") OR TITLE-ABS-KEY("questionnaire") OR TITLE-ABS-KEY("index") OR TITLE-ABS-KEY("battery") OR TITLE-ABS-KEY("checklist") OR TITLE-ABS-KEY("survey") OR TITLE-ABS-KEY("profile") OR TITLE-ABS-KEY("evaluation") OR TITLE-ABS-KEY("appraisal") OR TITLE-ABS-KEY("measurement") OR TITLE-ABS-KEY("rating") OR TITLE-ABS-KEY("metrics") OR TITLE-ABS-KEY("validation") OR TITLE-ABS-KEY("technique") OR TITLE-ABS-KEY("method") OR TITLE-ABS-KEY("approach")) | 48,316,560 |
| #4 Stroke | (TITLE-ABS-KEY("stroke") OR TITLE-ABS-KEY("strokes") OR TITLE-ABS-KEY("cerebrovascular accident") OR TITLE-ABS-KEY("cerebrovascular accidents") OR TITLE-ABS-KEY("cerebral stroke") OR TITLE-ABS-KEY("cerebral strokes") OR TITLE-ABS-KEY("stroke cerebral") OR TITLE-ABS-KEY("strokes cerebral") OR TITLE-ABS-KEY("cerebrovascular apoplexy") OR TITLE-ABS-KEY("apoplexy cerebrovascular") OR TITLE-ABS-KEY("brain vascular accident") OR (TITLE-ABS-KEY("blood vessels") OR TITLE-ABS-KEY("vascular") OR TITLE-ABS-KEY("neovascularization, pathologic") OR TITLE-ABS-KEY("pathologic neovascularization") OR TITLE-ABS-KEY("vascularisation") OR TITLE-ABS-KEY("vascularization") OR TITLE-ABS-KEY("vascularisations") OR TITLE-ABS-KEY("vascularise") OR TITLE-ABS-KEY("vascularised") OR TITLE-ABS-KEY("vascularities") OR TITLE-ABS-KEY("vascularitis") OR TITLE-ABS-KEY("vascularity") OR TITLE-ABS-KEY("vascularizations") OR TITLE-ABS-KEY("vascularize") OR TITLE-ABS-KEY("vascularized") OR TITLE-ABS-KEY("vascularizes") OR TITLE-ABS-KEY("vascularizing") OR TITLE-ABS-KEY("vasculars")) AND TITLE-ABS-KEY("accidents brain") OR TITLE-ABS-KEY("cerebrovascular strokes") OR TITLE-ABS-KEY("strokes cerebrovascular") OR TITLE-ABS-KEY("stroke cerebrovascular") OR TITLE-ABS-KEY("acute stroke") OR TITLE-ABS-KEY("stroke acute")) | 24,997 |
|  | #1 and #2 and #3 and #4 | 255 |
| **CINAHL** | **Search strategy** |  |
| #1 Deglutition Disorders | ("deglutition disorders" OR "disorders deglutition" OR "dysphagia" OR "deglutition disorder" OR "swallowing disorders" OR "swallowing disorder" OR "oropharyngeal dysphagia" OR "dysphagia oropharyngeal" OR "esophageal dysphagia" OR "dysphagia esophageal" OR "deglutition" OR "deglutitions" OR "swallowing" OR "swallowings") | 1,187 |
| #2 Sensitivity/ specificity | ("predictive value of tests" OR "predictive values of tests" OR "predictive value of test" OR "negative predictive value" OR "negative predictive values" OR "sensitivity and specificity" OR ("specificity" AND "sensitivity") OR ("sensitivity" AND "specificity") OR "sensitivity" OR "specificity" OR "diagnosis" OR "diagnoses" OR ("diagnoses" AND "examinations") OR ("examination" AND "diagnoses") OR "antemortem diagnosis" OR "postmortem diagnosis" OR "validity" OR "accuracy" OR "differential diagnosis" OR "differential diagnoses" OR "diagnostic errors" OR "diagnostic error" OR "diagnostic blind spots" OR "misdiagnoses") | 39,717 |
| #3 Scale | ("scale" OR "tool" OR "assessment" OR "test" OR "examination" OR "screen" OR "instrument" OR "questionnaire" OR "index" OR "battery" OR "checklist" OR "survey" OR "profile" OR "evaluation" OR "appraisal" OR "measurement" OR "rating" OR "metrics" OR "validation" OR "technique" OR "method" OR "approach") | 132,335 |
| #4 Stroke | ("stroke" OR "strokes" OR "cerebrovascular accident" OR "cerebrovascular accidents" OR "cerebral stroke" OR "cerebral strokes" OR "stroke cerebral" OR "strokes cerebral" OR "cerebrovascular apoplexy" OR "apoplexy cerebrovascular" OR "brain vascular accident" OR (("blood vessels" OR "vascular" OR "neovascularization, pathologic" OR "pathologic neovascularization" OR "vascularisation" OR "vascularization" OR "vascularisations" OR "vascularise" OR "vascularised" OR "vascularities" OR "vascularitis" OR "vascularity" OR "vascularizations" OR "vascularize" OR "vascularized" OR "vascularizes" OR "vascularizing" OR "vasculars") AND "accidents brain") OR "cerebrovascular strokes" OR "strokes cerebrovascular" OR "stroke cerebrovascular" OR "acute stroke" OR "stroke acute") | 10,797 |
|  | #1 and #2 and #3 and #4 | 34 |

**Table S2. Risk-of-bias assessment of each study by QUADAS-2 questions**

| **Study** | **Quality evaluation results** | **Patients selection** | | | **Index test** | | **Reference Standard** | | **Flow and timing** | | | |
| --- | --- | --- | --- | --- | --- | --- | --- | --- | --- | --- | --- | --- |
|  |  | a1 | a2 | a3 | a1 | a2 | a1 | a2 | a1 | a2 | a3 | a4 |
| Pacheco Castilho 2021 | Medium | Y | Y | Y | Y | Y | N | U | U | Y | Y | Y |
| Umay 2018 | LOW | Y | N | Y | Y | Y | Y | Y | Y | Y | Y | U |
| Perry 2001 | LOW | Y | Y | Y | Y | U | N | U | U | N | U | Y |
| Simpelaere 2023 | LOW | Y | Y | Y | Y | Y | N | U | U | N | U | Y |
| Immovilli 2021 | HIGH | Y | Y | Y | Y | Y | Y | Y | Y | Y | Y | Y |
| Umay 2018 | HIGH | Y | Y | Y | Y | Y | Y | Y | Y | Y | Y | Y |
| Gandolfo 2019 | LOW | Y | Y | Y | Y | U | N | U | Y | N | U | Y |
| Sherman 2018 | HIGH | Y | Y | Y | Y | Y | U | Y | Y | N | Y | Y |
| Warnecke 2017 | HIGH | Y | Y | Y | Y | Y | Y | Y | Y | Y | Y | N |
| Behera 2018 | Medium | U | Y | Y | U | Y | Y | Y | Y | Y | Y | Y |
| Edmiaston 2014 | Medium | U | Y | Y | Y | Y | Y | Y | Y | Y | Y | Y |
| Somasundaram 2014 | HIGH | Y | Y | Y | Y | Y | Y | Y | Y | Y | Y | Y |
| Schrock 2011 | LOW | U | N | Y | N | Y | Y | Y | Y | Y | Y | Y |
| Kopey 2011 | LOW | U | Y | Y | U | Y | Y | Y | Y | Y | Y | N |
| Antonios 2010 | HIGH | Y | Y | Y | Y | Y | Y | Y | Y | Y | Y | Y |
| Bravata 2009 | Medium | U | Y | Y | U | Y | U | Y | Y | Y | U | Y |
| Martino 2008 | Medium | Y | Y | U | Y | Y | Y | Y | Y | Y | Y | Y |
| Cummings 2015 | Medium | U | Y | Y | Y | Y | U | Y | Y | Y | U | Y |
| Benfield 2021 | LOW | Y | Y | Y | Y | Y | Y | Y | Y | Y | Y | N |
| Toscano 2018 | HIGH | Y | Y | Y | Y | Y | Y | Y | Y | Y | Y | Y |
| Rofes 2014 | Medium | Y | Y | Y | Y | Y | Y | Y | Y | Y | Y | U |

Y: Yes; N:No; U:unclear.

**Table S3.The accuracy performance of all the studies.**

| **Study** | **Sensitivity** | **Sensitivity 95%CI** | **Specifcity** | **Specifcity 95%CI** | **PPV** | **NPV** | **TP** | **FP** | **TN** | **FN** | **accuracy** |
| --- | --- | --- | --- | --- | --- | --- | --- | --- | --- | --- | --- |
| Pacheco Castilho 2020 | 0.86 | 0.75 - 0.93 | 0.41 | 0.29 - 0.54 | 0.44 | 0.84 | 33.54 | 12.39 | 8.61 | 5.46 | 0.7025 |
| Umay 2018 | 0.97 | 0.92 - 0.99 | 0.72 | 0.63 - 0.79 | 0.74 | 0.8 | 74.69 | 10.08 | 25.92 | 2.31 | 0.890353982 |
| Simpelaere 2023 | 0.97 | 0.94 - 0.99 | 0.9 | 0.85 - 0.93 | 0.92 | 0.96 | 129.98 | 6.6 | 59.4 | 4.02 | 0.9469 |
| Perry 2001 | 0.72 | 0.59 - 0.82 | 0.68 | 0.55 - 0.79 | 0.5 | 0.97 | 10.8 | 12.16 | 25.84 | 4.2 | 0.691320755 |
| Umay 2018 | 1 | 0.97 - 1.00 | 0.95 | 0.90 - 0.98 | 0.9 | 1 | 36 | 4.2 | 79.8 | 0 | 0.965 |
| Immovilli 2020 | 0.73 | 0.64 - 0.80 | 0.89 | 0.82 - 0.94 | 0.65 | 1 | 48.18 | 4.73 | 38.27 | 17.82 | 0.793119266 |
| Gandolfo 2019 | 0.67 | 0.61 - 0.73 | 0.95 | 0.92 - 0.97 | 0.9 | 0.83 | 50.25 | 8.7 | 165.3 | 24.75 | 0.865662651 |
| Sherman 2018 | 0.37 | 0.30 - 0.45 | 0.94 | 0.89 - 0.97 | 0.81 | 0.68 | 22.2 | 5.22 | 81.78 | 37.8 | 0.707346939 |
| Warnecke 2017 | 0.93 | 0.86 - 0.97 | 0.56 | 0.46 - 0.65 | 0.75 | 0.95 | 65.1 | 13.2 | 16.8 | 4.9 | 0.819 |
| Behera 2018 | 0.69 | 0.63 - 0.75 | 0.93 | 0.89 - 0.96 | 0.77 | 0.9 | 40.02 | 11.69 | 155.31 | 17.98 | 0.868133333 |
| Edmiaston 2014 | 0.94 | 0.90 - 0.96 | 0.66 | 0.60 - 0.72 | 0.71 | 0.93 | 99.64 | 40.46 | 78.54 | 6.36 | 0.791911111 |
| Somasundaram 2014 | 0.9 | 0.81 - 0.95 | 0.5 | 0.38 - 0.62 | 0.47 | 0.71 | 27 | 18.5 | 18.5 | 3 | 0.679104478 |
| Schrock 2011 | 0.95 | 0.92 - 0.97 | 0.55 | 0.49 - 0.61 | 0.5 | 0.95 | 86.45 | 86.4 | 105.6 | 4.55 | 0.678621908 |
| Kopey 2011 | 0.21 | 0.16 - 0.27 | 0.98 | 0.95 - 0.99 | 0.88 | 0.72 | 15.12 | 3.02 | 147.98 | 56.88 | 0.731390135 |
| Antonios 2010 | 0.89 | 0.83 - 0.93 | 0.85 | 0.78 - 0.90 | 0.77 | 0.93 | 48.06 | 14.4 | 81.6 | 5.94 | 0.8644 |
| Bravata 2009 | 0.29 | 0.21 - 0.38 | 0.84 | 0.76 - 0.90 | 0.5 | 0.68 | 11.02 | 10.08 | 52.92 | 26.98 | 0.633069307 |
| Martino 2008 | 0.91 | 0.87 - 0.94 | 0.66 | 0.61 - 0.71 | 0.76 | 0.93 | 23.66 | 14.28 | 27.72 | 2.34 | 0.755588235 |
| Cummings 2015 | 0.89 | 0.77 - 0.95 | 0.9 | 0.78 - 0.96 | 0.83 | 0.93 | 16.02 | 3.1 | 27.9 | 1.98 | 0.896326531 |
| Benfield 2021 | 0.46 | 0.29-0.63 | 0.83 | 0.52–0.98 | 0.89 | 0.35 | 16 | 2 | 10 | 19 | 0.55 |
| Toscano 2018 | 1 | 0.88-1.00 | 0.77 | 0.60-0.95 | 0.85 | 1 | 28 | 5 | 17 | 0 | 0.9 |
| Rofes 2014 | 0.94 | 0.87–0.98 | 0.88 | 0.50–0.99 | 0.98 | 0.7 | 90 | 12 | 88 | 12 | 0.89 |

**
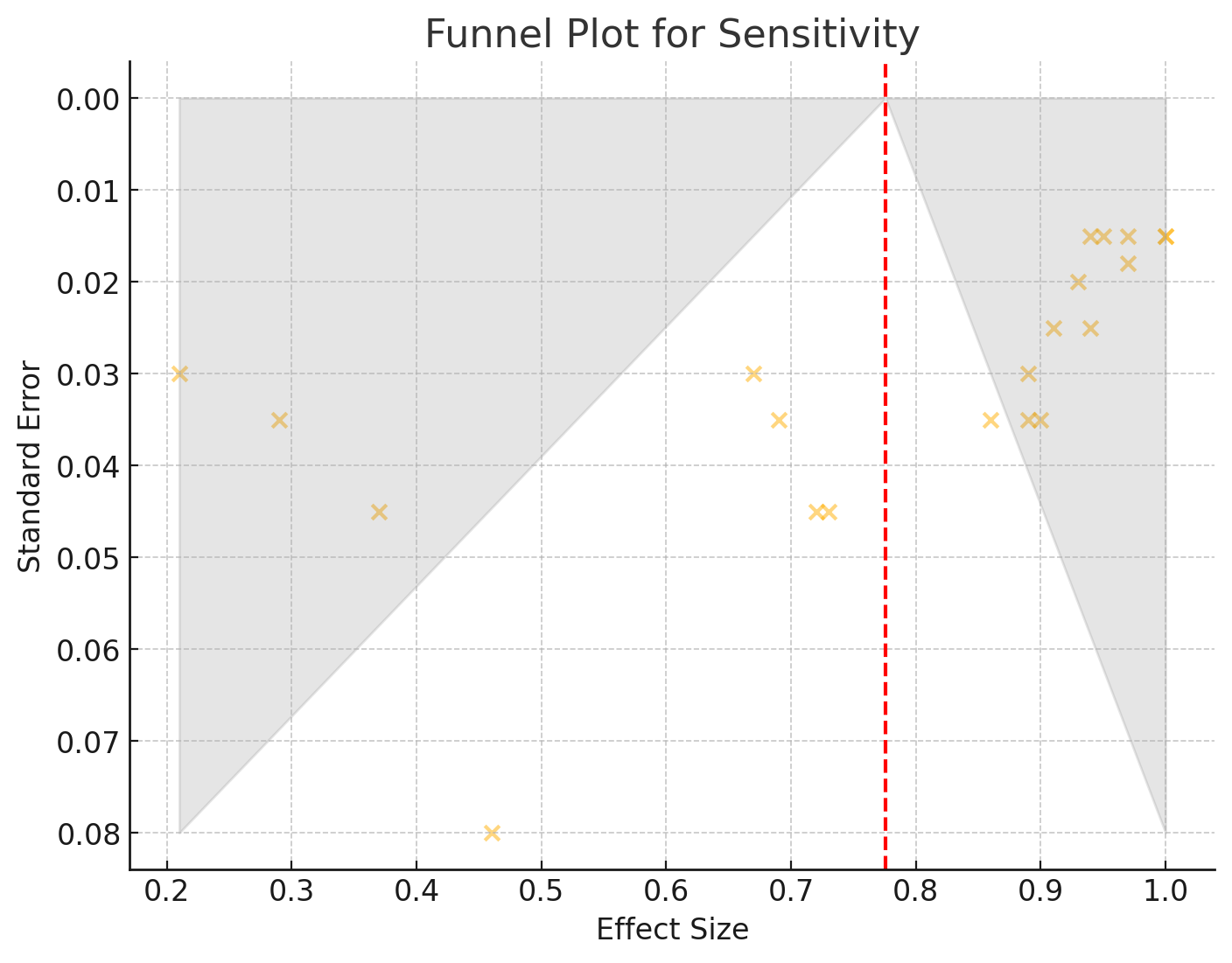
**

**Figure S1. Publication bias of sensitivity as the outcome measure.**

**
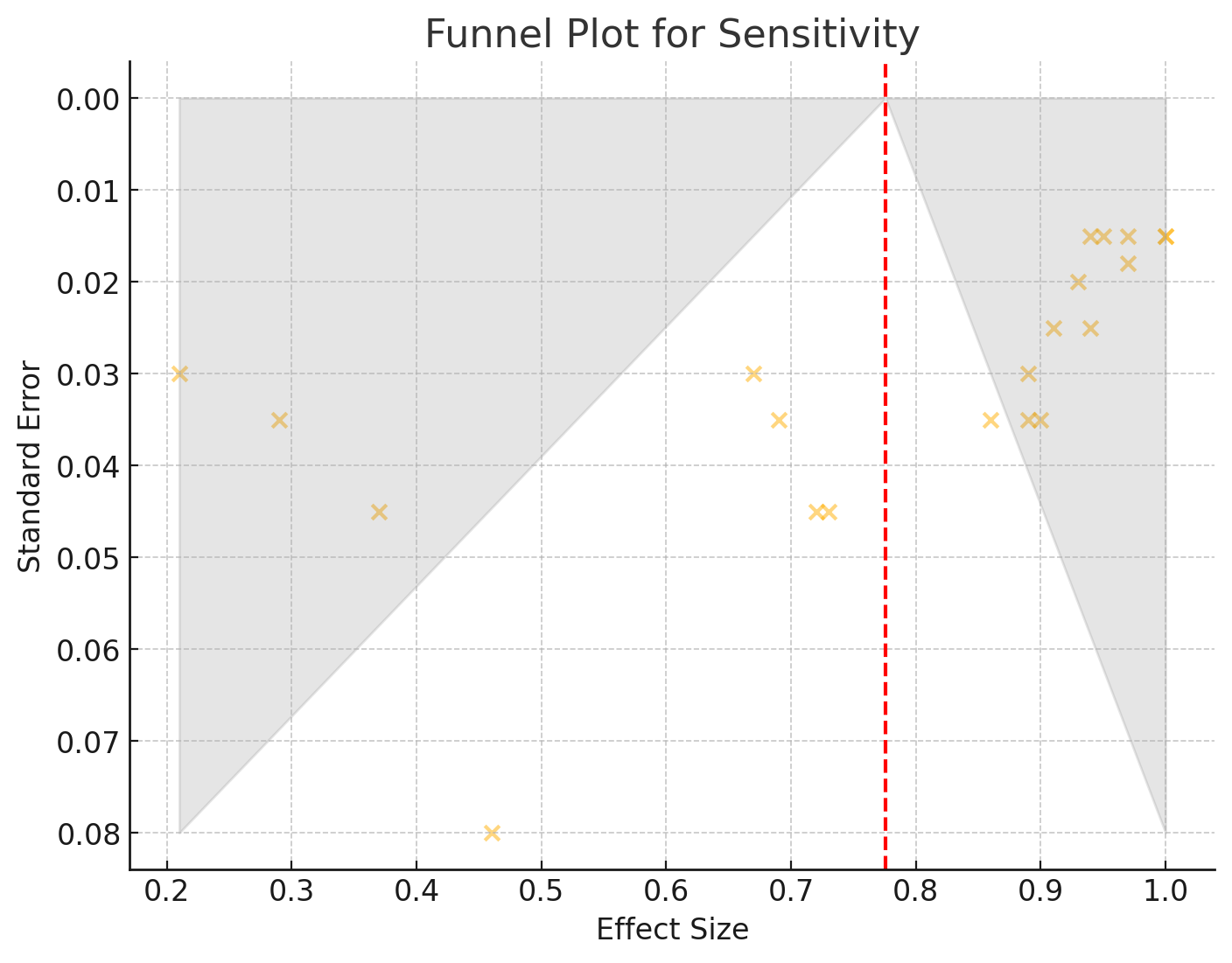
**

**Figure S2. Publication bias of specificity as the outcome measure.**

**
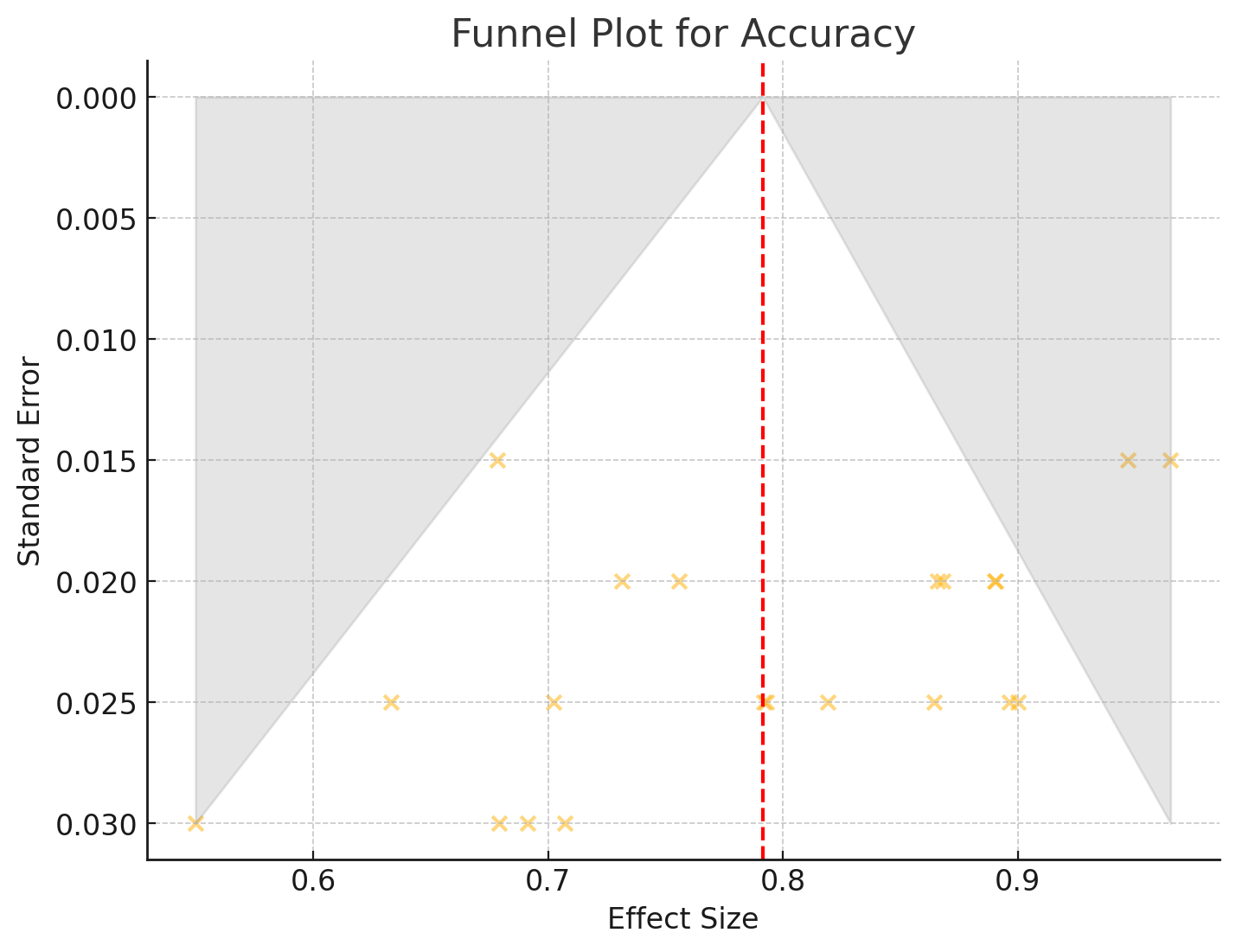
**

**Figure S3. Publication bias of accuracy as the outcome measure.**

**Table S4. QUADAS-2**

| **DOMAIN 1: PATIENT SELECTION**  **A. Risk of Bias**   \| Describe methods of patientselection: \| \| --- \| | | |
| --- | --- | --- | --- |
| ❖ Was a consecutive or random sample of patients enrolled? ❖ Was a case-control design avoided?  ❖ Did the study avoid inappropriate exclusions? | | Yes/No/Unclear Yes/No/Unclear Yes/No/Unclear |
| **Could the selection of patients have introduced bias?**  **B. Concerns regarding applicability** | **RISK: LOW/HIGH/UNCLEAR** | |
| \| Describe included patients (prior testing, presentation, intended use of index test and setting)**:** \| \| --- \|   **Is there concern that the included patients do not match CONCERN: LOW/HIGH/UNCLEAR**  **the review question?** | | |

| **DOMAIN 2: INDEX TEST(S)**  **If more than one index test was used, please complete for each test.**  **A. Risk of Bias**   \| Describe the index test and how it was conducted and interpreted: \| \| --- \| | | |
| --- | --- | --- | --- |
| ❖ Were the index test results interpreted without  knowledge of the results of the reference standard? | | Yes/No/Unclear |
| ❖ If a threshold was used, was it pre-specified?  **Could the conductor interpretation of the index test have introduced bias?** | Yes/No/Unclear  **RISK: LOW /HIGH/UNCLEAR** | |
| **B. Concerns regarding applicability**  **Is there concern that the index test, its conduct, or interpretation differ from the review question?** | **CONCERN: LOW /HIGH/UNCLEAR** | |

| **DOMAIN 3: REFERENCE STANDARD**  **A. Risk of Bias**   \| Describe the reference standard and how it was conducted and interpreted: \| \| --- \| | | |
| --- | --- | --- | --- |
| ❖ Is the reference standard likely to correctly classify the target condition?  ❖ Were the reference standard results interpreted without | | Yes/No/Unclear  Yes/No/Unclear |
| knowledge of the results of the index test?  **Could the reference standard, its conduct, or its interpretation have introduced bias?**  **B. Concerns regarding applicability**  **Is there concern that the target condition as defined by the reference standard does not match the review question?** | **RISK: LOW /HIGH/UNCLEAR**  **CONCERN: LOW /HIGH/UNCLEAR** | |

| **DOMAIN 4: FLOW AND TIMING**  **A. Risk of Bias**   \| Describe any patients who did not receive the index test(s) and/or  reference standard or who were excluded from the 2x2 table (refer to flow diagram)  Describe the time interval and any interventions between index test(s) and  reference standard: \| \| --- \| | |
| --- | --- | --- |
| ❖ Was there an appropriate interval between index test(s) and reference standard? | Yes/No/Unclear |
| ❖ Did all patients receive a reference standard? Yes/No/Unclear  ❖ Did patients receive the same reference standard? Yes/No/Unclear  ❖ Were all patients included in the analysis? Yes/No/Unclear  **Could the patient flow have introduced bias? RISK: LOW /HIGH/UNCLEAR** | |
